# Development of a managed clinical network for children’s palliative care – a qualitative evaluation

**DOI:** 10.1186/s12904-021-00712-7

**Published:** 2021-01-22

**Authors:** Andrew Papworth, Lorna Fraser, Jo Taylor

**Affiliations:** 1 Martin House Research Centre, Department of Health Sciences, Seebohm Rowntree Building, University of York, Heslington, York, YO10 5DD UK; 2 Department of Health Sciences, University of York, York, UK

**Keywords:** Managed clinical network, Children’s palliative care

## Abstract

**Background:**

Consistent evidence suggests that children’s palliative care is not equitable and managed clinical networks (MCNs) have been recommended as a solution. This study explored the perspectives of health professionals involved in the development of a children’s palliative care MCN, with an aim to identify barriers and enablers of successful implementation.

**Methods:**

Thematic analysis of semi-structured interviews and focus groups with 45 healthcare staff with a role in developing the MCN or in the delivery of children’s palliative care (September 2019–March 2020).

**Results:**

The study explored health professionals’ perceptions of the MCN features that had helped to formalise governance processes, establish training and networking opportunities, standardise practice, and improve collaboration between organisations. These include the funded MCN co-ordinator, committed individuals who lead the MCN, and a governance structure that fosters collaboration. However, the MCN’s development was impeded by cross-cutting barriers including limited funding for the MCN and children’s palliative care more generally, no shared technology, lack of standards and evidence base for children’s palliative care, and shortage of palliative care staff. These barriers impacted on the MCN’s ability to improve and evaluate palliative care provision and affected member engagement. Competing organisational priorities and differences between NHS and non-NHS members also impeded progress. Training provision was well received, although barriers to access were identified.

**Conclusions:**

Key features of children’s palliative care can act as barriers to developing a managed clinical network. Managing expectations and raising awareness, providing accessible and relevant training, and sharing early achievements through ongoing evaluation can help to sustain member engagement, which is crucial to a network’s success.

**Supplementary Information:**

The online version contains supplementary material available at 10.1186/s12904-021-00712-7.

## Background

Palliative care for children is defined by the World Health Organisation (WHO) as “the active total care of the child’s body, mind and spirit, and also involves giving support to the family” [1]. It is a comparatively new medical specialty with a small and specialised workforce in the UK [2], which includes third sector providers as well as NHS services [3], and covers a broad spectrum of diseases within a relatively small population [4].

The development of children’s palliative care services in the UK was unplanned and not centrally coordinated [5–7, 8]. As a result, provision may be “good in some areas, [but] in others it is generally unclear who is providing what (if anything), and to whom, thus leading to substantial unmet needs” [5]. Evidence consistently shows this inequality [3, 5, 9, 10], with patchy geographical distribution of provision [3, 11], differing structures, services and models of care among providers [5, 12, 13], and a lack of collaboration and coordination between these organisations [3, 5, 12]. Establishing managed clinical networks (MCNs) [14] for children’s palliative care has been proposed to address these issues [15], and to ensure specialist palliative care is available to those who need it [16, 17].

MCNs are defined as a network of health staff and organisations working together to make sure that high quality, clinically effective services are fairly distributed [18]. They have been established for various care pathways and medical specialties in the UK and other countries [19]. Unlike informal professional networks which are organic by nature, the key feature of MCNs is that they are formal and managed entities in which the emphasis “shifts from buildings and organisations to services and patients” [14], often requiring members to “‘surrender sovereignty’ to achieve shared objectives” [20].

The current evidence about MCNs suggests they lead to improved care processes and clinical outcomes. A recent systematic review identified a lack of high-quality studies examining effectiveness [19], but also identified factors associated with positive outcomes. These are summarised in Table 1.

**Table 1:** Features of successful clinical networks – enablers and barriers

**Enablers of managed clinical network success**	
Sufficient resources – funding, administration and human (staffing)	
Availability of information and communication technologies	
A bottom-up, locally driven approach to implementation	
A positive, trusting culture where networks are seen as desirable and perceived to be necessary	
The norms and values of the network are compatible with those of the organisations involved	
Strong leadership	
Inclusive membership	
Engagement at different levels of the healthcare system	
Evidence based work plans and projects that address issues identified by network members	
Supportive policy environments	
**Barriers to managed clinical network success**	
Lack of funding and resources	
Tension, distrust and competition (particularly over resources) between network members	
An imbalance of power between network members resulting in competition for resource	
Poor communication and unwillingness to collaborate	
Lack of confidence in the ability of network leaders and managers	
Lack of representation of key stakeholders in certain contexts (e.g. rural interests)	
Poor record keeping and documentation	
A top-down approach of network implementation	

Source: Adapted from Brown et al. (2016) [19]

There is no published evidence about the implementation of MCNs for children’s palliative care. An understanding of how the distinct features of this specialism may influence the implementation of MCNs is needed. This study focused on a regional MCN for children’s palliative care (‘the MCN’) in England and aimed to identify the key barriers to and enablers for its successful implementation.

## Methods

Qualitative methods were employed to explore meanings, experiences and beliefs about the MCN among individuals with a role in developing the MCN and staff who work in the MCN’s member organisations. The study was designed with two phases of data collection, both aimed at understanding the perceptions of individuals with different relationships to the MCN. The two phases are discussed further in the sample and recruitment section below.

The study was underpinned by two theories that offered a useful lens through which to examine different aspects of implementation of the MCN. The first was Socio-technical Systems Theory (SST), which is based on the idea that the performance of an organisational system can only be understood by bringing ‘social’ and ‘technical’ aspects of the organisation together and viewing a system as being made up of interacting sub-systems with their own unique infrastructures, cultures and goals [21–23].

The second theory was Normalization Process Theory (NPT), which consists of four core constructs that represent the different work of implementation (see Table 2) [24–27]. These two theories provided a useful framework through which to assess the work that has been carried out so far and to understand how the identified barriers relate to the different work associated with implementation.

**Table 2:** Normalisation Process Theory constructs [24–27]

Construct name	Construct definition
Coherence	The **sense-making work** that people do to differentiate the MCN from existing practice, to develop a shared understanding of the Network’s goals, and to understand what this means for their own individual practice and what the benefits are.
Cognitive participation	The **relational work** that people do to initiate the new set of practices associated with the MCN, and to build commitment and engagement from other members to ensure new ways of working are legitimised and sustained.
Collective action	The **operational work** that people do to implement a set of practices and build confidence in these, for example developing ways to share patient information or specialist palliative care resources between organisations.
Reflexive monitoring	The **appraisal work** that people do individually and together to understand the ways that the MCN affects them and others around them (e.g. children and their families), and to modify new processes to make them workable in practice.

### The MCN

**Fig. 1:**
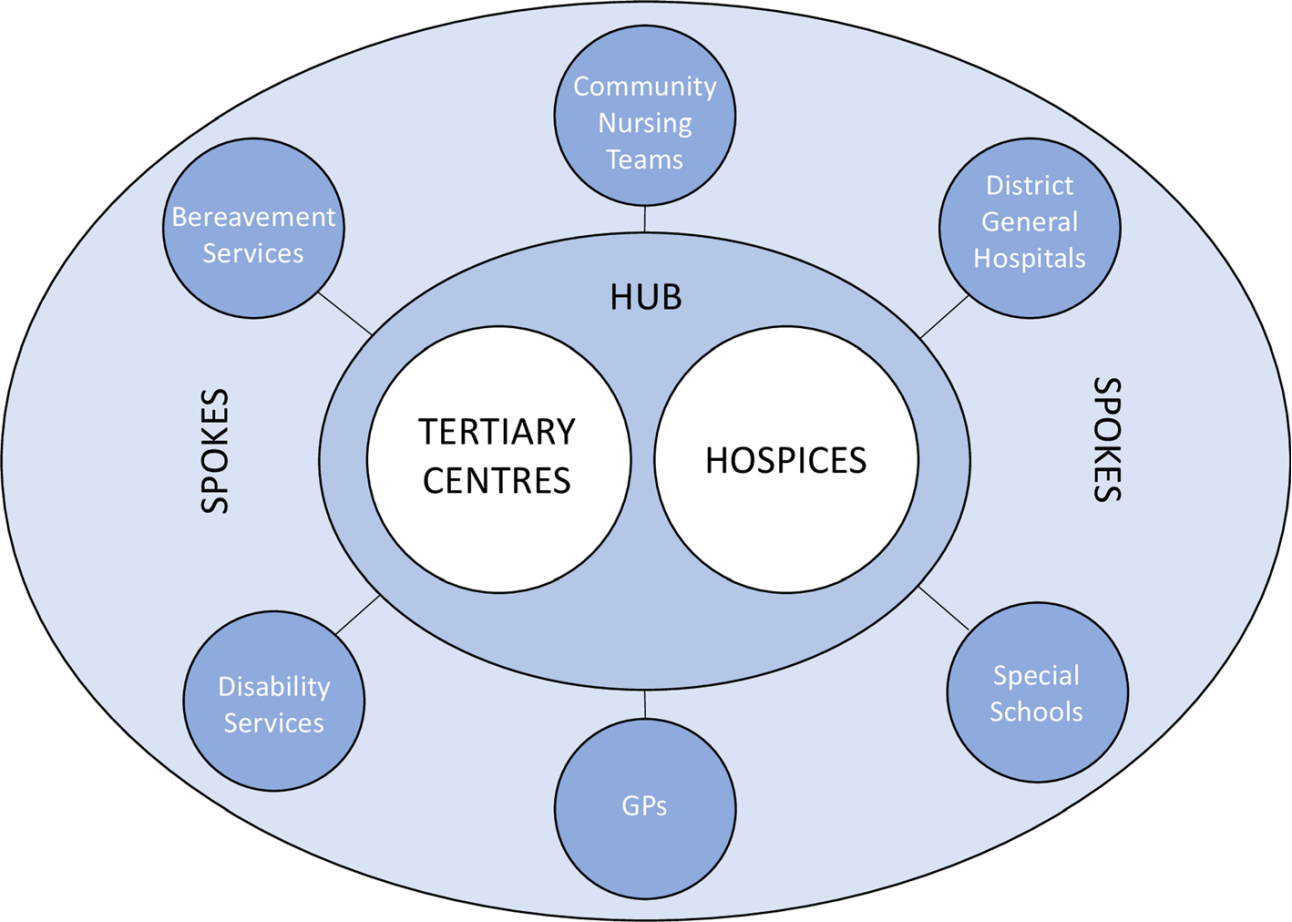
Schematic of the hub-and-spoke model used by the Network. Source: Authors

The MCN investigated is in a large geographical region of England with a mix of dense urban and sparsely populated rural localities. The region is served by a small number of paediatric palliative care clinicians, children’s hospices, tertiary centres and specialist nursing teams. An informal professional network has existed for a number of years, but at the time of the study, this was transitioning into an MCN. Its aims include the development of palliative medicine teams in the local children’s hospitals and the creation of 24/7 community nursing services in the region. The MCN has a hub-and-spoke model [28], as seen in Fig. 1. The hubs are intended to act as ‘centres of excellence’ with all organisations working together to share information, education and clinical guidelines.

### Sample and recruitment

Phase 1 aimed to explore the perspectives of individuals involved in the MCN’s core internal structures, which consisted of an executive committee and two steering groups. All individuals with a role in these structures were invited to take part (*n* = ~ 35).

Phase 2 focused on participants who worked in hub and spoke organisations but were not involved in the MCN’s internal structures. Organisations were purposively sampled to ensure representation from different services (both hub and spoke), with a particular focus on those not already represented in phase 1 – which included those on the MCN’s periphery, either geographically or due to limited engagement in the MCN – as these had not been sampled in the first phase of data collection. Convenience and snowballing strategies were used to identify potential participants. We expected to recruit 20–30 participants in phase 2.

All participants received a study information sheet and provided either verbal recorded, email or written consent prior to participation.

### Data collection

In phase 1 we conducted semi-structured (face-to-face or telephone) interviews with members of the executive committee. The membership of the MCN’s two steering groups is quite fluid, so it was felt that focus groups would be the most appropriate method to understand the varied perceptions of those that are part of these groups [29]. In phase 2 we used semi-structured (telephone) interviews.

The topic guides (see supplementary information) for both phases were designed with reference to SST, NPT and existing knowledge about the MCN, and focused mainly on implementation work, barriers, enablers and achievements [21, 23–27]. The topic guide for phase 2 also drew on the initial analysis of phase 1 data.

Interviews lasted approximately 20–30 min and were conducted by AP. AP and JT facilitated the focus groups, which were around 60 min long. Data were collected between September 2019 and March 2020. Data collection ceased when no new information was being collected that would alter our understanding of the aims of the study. Focus groups and interviews were audio-recorded and all but one were transcribed intelligent verbatim for analysis. Extensive notes were made on the final interview, which could not be transcribed due to poor recording quality.

### Data analysis

Drawing on approaches used in other implementation research [30], data were analysed thematically [31] using an initial inductive coding process informed by the study aims to identify themes (i.e. barriers and enablers of implementation), which were then mapped onto the SST framework (see Table 3). Phase 1 data were analysed first, informing the coding and analysis of phase 2 data. NVivo version 12 [32] was used to support the analysis.

**Table 3:** Analytical process

Analytical step	What we did	Who was involved
Step 1: ‘Inductive coding’	Inductive coding of words, sentences and phrases that pertained to factors influencing MCN implementation	AP
Step 2: ‘Identification’	Identification of themes, which for this study were ‘factors’, or barriers and enablers, from the inductive codes	AP and JT
Step 3: ‘Refinement’	Development and refinement of themes, exploring how phase 1 and phase 2 participants contributed to these, and exploring of patterns and relationships within these themes	AP and JT with input from LF
Step 4: ‘Mapping’	Mapping of final themes to STS framework	AP, JT and LF

## Results

Thirty-three participants from 15 organisations took part in phase 1. Fourteen of the 18 executive committee members took part in interviews and 19 others took part in the two focus groups. The majority of the participants in this phase were in management roles as a result of the make-up of the MCN’s internal structures. Twelve participants from 10 organisations took part in phase 2 (total 45 participants from 20 organisations (see Tables 4 and 5)). The numbers in phase 2 were lower than expected; some invited participants excluded themselves because they felt they didn’t know enough about the MCN and we reached data saturation earlier than expected.

**Table 4:** Study participant numbers by organisation type

Type of organisation	Hub organisations		Spoke organisations		Total
	*NHS*	*Non-NHS*	*NHS*	*Non-NHS*	
Phase 1	5	18	8	2	33
Phase 2	3	5	3	1	12
Total	8	23	11	3	45

**Table 5:** Study participant numbers by professional role

Type of role	Clinical	Nursing	Management	Other	Total
Phase 1	9	8	15	1	33
Phase 2	3	3	4	2	12
Total	12	11	19	3	45

**Fig. 2:**
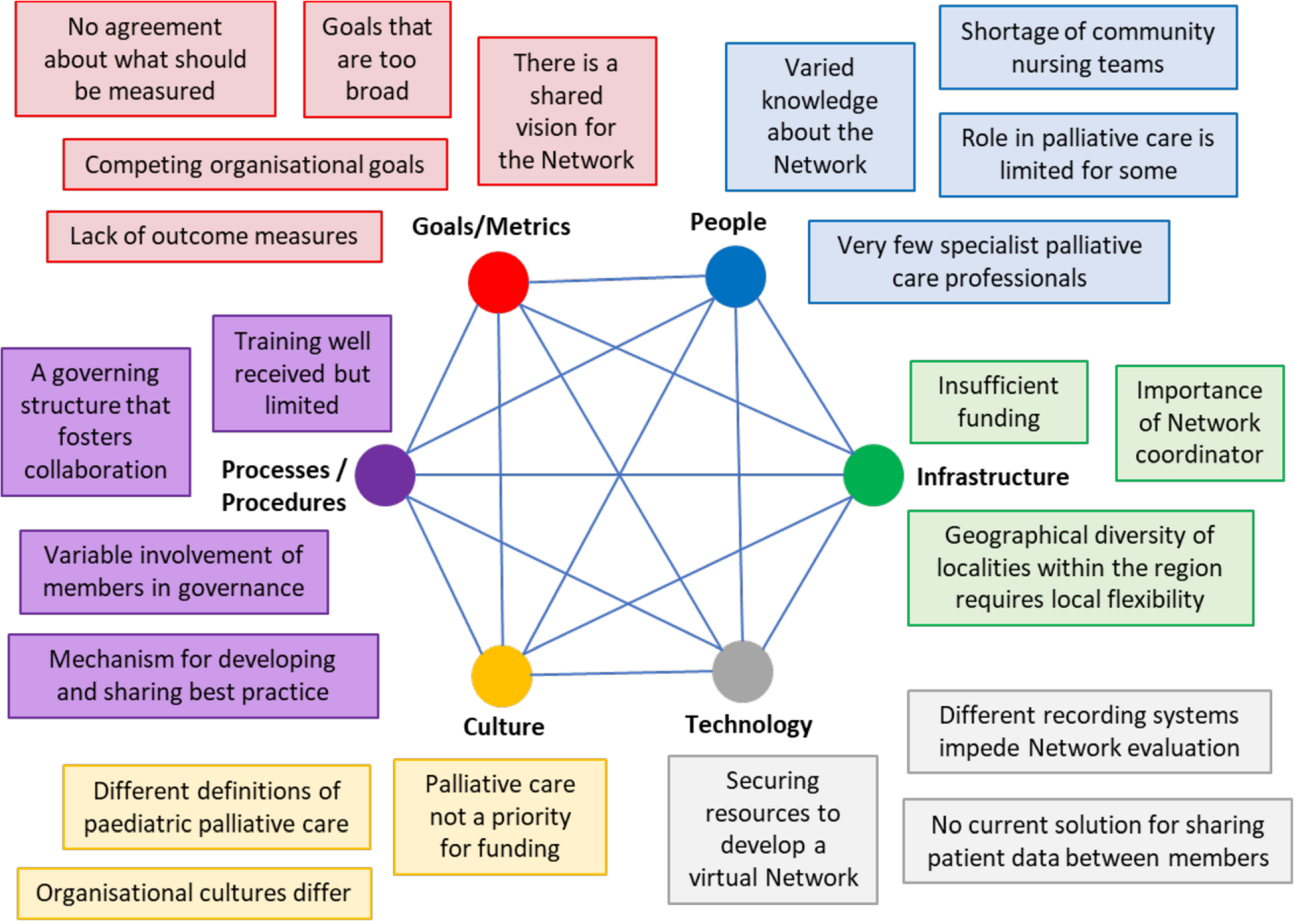
Factors affecting implementation of the Network. Source: Adapted from Davis et al. 2014 [23]

Figure 2 shows how the themes were mapped onto the SST framework, summarises the identified factors affecting MCN implementation, and demonstrates their connected nature. The results are not presented in order of importance, but are structured around the SST, with each section of the framework presented in turn. The factors are described further below, using participant quotations to illustrate key points and relationships between factors. Additional indicative quotes are included in Table 6.

**Table 6:** Additional participant quotations mapped to themes and the SST framework

SST Theme		Indicative quotes
Goals and metrics	Shared vision for the MCN	“If we have a completely effective managed clinical network, I think we should be able to offer all children across the region, whatever their diagnosis, whatever their age, wherever they live, access to good palliative care.” (Phase 1, participant #2) “I imagine that it’s the standardised care and access to all children around the region, so they get good palliative care input and that it’s not a postcode lottery type thing, and everybody is doing similar things, but I don’t actually know what their [the Network] aims and objectives are (Phase 2, participant #47) “It highlights those areas and provides a bigger lever to [say a particular region is] failing [its] children by not funding [this] adequately.” (Phase 2, participant #39)
	Lack of outcome measures and measurement of outcomes	“Hearing things anecdotally … we can’t prove that … It doesn’t make any difference how many people tell me how much more confident they are, or how [many] families give us positive feedback, unless we can measure it.” (Phase 1, participant #2) “If you’ve got a diabetes network, you manage everybody’s HbA1c [and] that’s what trust boards and people [work towards, but] we can’t give that.” (Phase 1, focus group 2 participant) “Even things that we could measure, every organisation is measuring it in a different way. The people who do measure it aren’t always prepared to share that data with the other groups … There’s different people asking for different data, and it’s very unclear what data it is we’re supposed to be collecting.” (Phase 1, participant #2) “We were asked to do a data collection exercise … it was just a bit confused and we piloted it but other services didn’t and there was a suggestion that if everybody didn’t do it then the research wouldn’t really have much value” (Phase 2, participant #36)
	Having goals that are too broad	“I think, in retrospect, [you look at the Strategy] now and think, is it too broad? Are we trying to answer too many problems? Are we trying to fix too much in our first strategy?” (Phase 1, participant #5)
	Competing organisational goals	“It’ll be a lot clearer for trusts and commissioners as to what’s expected, and that makes it easier for us to bargain about services, about what we need to deliver things.” (Phase 1, participant #21)
People	Varied knowledge about the Network	“I know it’s a bunch of people trying to coordinate the palliative care services across hospice, part hospital, and how to transition all the kids out into the adult arena and it provides updates” (Phase 2, participant #39) “I’m aware of what the plan is in the sense that the network would like to have 24/7 a region-wide specialist palliative care on-call service.” (Phase 2, participant #35) “We are supposed to be a hub, I have no idea what that means. I am kind of being asked to sign up to something that I don’t really know what it means.” (Phase 1, participant #3) “Well, I know that they run educational days … I think they’ve got a website as well …the ReSPECT document … they’ve been working on that together. I don’t know otherwise I have to say” (Phase 2, participant #47) “It’s difficult for me to say whether it’s successful but all I can say, from my perspective is, there’s no impact on the group as far as I can see and if there has been and I don’t know about it, then it’s not been communicated to me.” (Phase 2, participant #36) “I did attend a few of the network meetings … which fizzled out a bit and it always a bit hit and miss, and people didn’t attend. That’s why I’ve pulled away from it over the last few years. I’m not sure I know much about it.” (Phase 2, participant #40)
	Role in palliative care is limited for some	“The people that were attending [Network meetings] were involved in palliative care, the main part of their job. It just isn’t in our role … I think that’s why I found it difficult because I just felt it was just a fraction of our work, the palliative side of it.” (Phase 2, participant #40)
	Shortage of palliative care staff	“The children’s community nurses were telling me that they didn’t feel happy to manage these children, without somebody [a specialist] they could call. They are saying they don’t do this very often, and they don’t feel experienced in doing it.” (Phase 1, participant #2) “Scaling up [specialist] services to actually provide [for] such a small number of children would be not very cost effective.” (Phase 1, participant #16) “24/7 specialist cover I think is another tricky issue because we haven’t got enough super specialists to go round.” (Phase 1, participant #6)
Infrastructure	Insufficient funding for the MCN	“This [should be] a long-term project. There is no way any of this is going to happen in a year [but] funding structures in the NHS and the government don’t allow for long term projects, and long-term investment.” (Phase 1, participant #16) “It’s funded very little, we hardly have any funding at all, so it’s really people doing it in their own time, because we all want to achieve the same things, more or less.” (Phase 1, participant #18) “They [hospices] see survival as being more important than, “Let’s try and deliver five strategic aims that are almost impossible to deliver in the next five years, and won’t be delivered in the next five years”. We need to focus on survival.” (Phase 1, participant #5)
	A challenging geography	“Because we’re quite a big region it can take nearly three hours to get from one end of it to the other, maybe longer. So if we’ve got meetings inevitably there are people that are having to take out quite a lot of time out of their day. And so sort of voluntary organisations, there’s a time, there’s a cost element attached to that as well. So I think that’s a major barrier.” (Phase 1, participant #10) “Different areas have different levels of need, so it’s about recognising that as well and tailoring it, I suppose, to the different areas, because there’s obviously areas that have got huge numbers of palliative care children with life-limiting conditions” (Phase 1, participant #18) “The geography for [particular locality in the region] has already changed and it will be all about integrated care systems and if the managed clinical network is not around integrated care systems then it’s behind the times already.” (Phase 1, participant #6)
	Importance of the Network coordinator	“I was contacted by a specialist in another tertiary centre about a patient from somewhere that she thought was my neck of the woods. [It] wasn’t, [but] I was able to [tell them to contact the coordinator]. Years ago, that would have taken a lot of trying to find that out.” (Phase 1, participant #21) “[We cannot] achieve a strategy that is…that ambitious with one member of staff.” (Phase 1, participant #3)
Technology	No current solution for sharing patient data	“So, when the child is in the hospital, it will flag up that they’re not for resuscitation. But the second they’re out of that, they’ll have to physically show a paper copy of it, because otherwise no-one knows it exists.” (Phase 1, participant #2) “It’s great in theory to have a 24/7 service that you want to provide a telephone service for, and that’s great, but if you can’t access the medical record of the person that you are giving advice to in the region then that’s going to be very limited in what you can actually achieve.” (Phase 1, participant #21) “My advanced nurse practitioners would be really nervous of … they are alright with their own cases because they know the children and they know they have got access to the notes, but doing that for somebody else’s children, they would be very nervous about that.” (Phase 1, participant #5)
	Different recording systems impede MCN evaluation	“A lot of the work that happens in community nursing teams and hospices is done on paper, paper and pen. We are not fully digital, we’re not even getting to the majority of being digital.” (Phase 1, participant #2)
Culture	Different definitions of paediatric palliative care	“I think, to solve the problem, we need to go right back to the beginning, and first of all clarify what are our definitions, what are we actually doing? Which of the people we’re trying to do this for, and for what period of their life?” (Phase 1, participant #2) “To progress in this conversation with the CCG leads, we’ve got willingness, we’ve got people coming to the table, we’ve got people round the table, then we need to be really clear about what our definitions are about which group of young people and which function we’re actually looking into” (Phase 1, participant #8)
	Palliative care not a priority for funding	“There’s lots going on out there that have got big budgets attached to them, and big teams, and I guess that’s where the focus goes. And I understand that, but it just means we have a very small voice as a collective, when we talk about children’s palliative care.” (Phase 1, participant #5) “In some ways, I feel that a lot of the hospitals across the region have almost got away without providing palliative care, even though it’s recommended by NHS, and it’s recommended by NICE, because they feel the hospices are picking it up.” (Phase 1, participant #2) “If the hospices were funded properly in the first place, we wouldn’t be in the position that we are in … it is about protecting your own organisation first and foremost” (Phase 1, participant #3) “[We need] some senior leadership saying, “No, actually, this is a priority, this is ring-fenced money to spend on children with palliative care needs.” (Phase 1, participant #16)
	Organisational cultures differ	“I think making it work is not an issue about money, it is an issue about culture. And I think one of the issues about culture is that in order to collaborate and work together you have got to be able to give stuff away, and if you are not prepared to do that you are never going to be able to move on.” (Phase 1, participant #3) “I think there’s always an element of that change might mean a threat to my organisation or to my job. I think networks by their very nature are about collaboration, coming together and often there’s difficulties around competition between individual organisations especially when it comes to funding.” (Phase 1, participant #10)
Processes and procedures	A governing structure that fosters collaboration	“Bringing people together and having organisations and individuals from organisations sat round a table, does just generally improve cooperation and working practices … raises awareness of how things are done, and improves the way people work together.” (Phase 1, participant #16) “I think it’s possibly that a lot of the activity is quite high level in talking to NHS England, having conversations at that level and it doesn’t feel like there is anything that’s influenced practice on the ground in any way, I would say.” (Phase 2, participant #36) “Our managed clinical network is struggling to show its effectiveness because of the difficulties with data collection and outcomes.” (Phase 1, participant #2) “I think that progress has been made, but I can’t tell you where we’re at … And I think a lot of it is still planning how it’s going to be implemented” (Phase 1, participant #18)
	Variable involvement of member organisations in governance	“We see a differing cast [each meeting] in terms of those individuals who attend. Some organisations have never been represented at the executive committee, some have been represented but by different people, at different times, at different levels of seniority.” (Phase 1, participant #5) “So some organisations have never been represented at the executive committee, some have been represented but by different people, at different times, at different levels of seniority.” (Phase 1, participant #2)
	A mechanism for developing and sharing best practice	“There will be variation in what [services] do, and how they do it. And so, implementing any kind of minimum standards and expectations could be difficult, because if I involve some [services] changing what they’re already doing, or stopping something, starting something new, which they might not want to do.” (Phase 1, participant #16) “The 24-h, seven day a week service that we would like to offer, I’ve been involved in that, and that feels like how would we like it to look, how would it work in practice, but we’re not ready to set that up yet.” (Phase 1, participant #18) “We have written a document for the 24/7 nursing model, which has been done a small clinical group, where it needs to be approved by commissioners, and funding identified to try and get the teams to be properly funded, so they can flex up to provide 24/7 care” (Phase 1, participant #15)
	Training that is well received but limited	“I got more of an understanding of how different professionals approach situations … I think in many ways my approach has … improved.” (Phase 2, participant #35) “It helps you grow with your own professional development and you definitely take it with you and wear it … they’re really important and if we could go all the time, we’d love to” (Phase 2, participant #45) “I’ve read the itineraries for this year’s conference and it’s just totally put me off really … because it is completely medical. There doesn’t seem to be any sharing about … actual patient care in the hospice.” (Phase 2, participant #37)

### Goals and metrics

Nearly all participants in both phases articulated a shared vision for the MCN: to achieve equitable and high-quality children’s palliative care provision and develop a 24/7 model of care. However, a small number raised concerns that the MCN’s goals were too broad, which one participant linked to the definition of palliative care adopted by the MCN:

*“[Our] definition […] talks about [providing services] from the point of recognition of a life-limiting condition all the way through to death and beyond. If you’re trying to provide that for everyone […] actually it’s a massive task.” (Phase 1, participant #2)*

Some participants in both phases acknowledged that they were most concerned with the care provision and future of their own organisation and didn’t want to compromise that to improve the MCN.

*“[We are focused on creating] a business model that’s financially sustainable […] so initiatives like the [MCN] can sometimes play second fiddle.” (Phase 1, participant #5)*

Others stated the MCN was a positive distraction as it helped children’s palliative care to remain on the agenda in their organisation.

*“[The MCN gives] a degree of status that means it’s difficult for both the trust and the commissioners to ignore.” (Phase 1, participant #21)*

The lack of a standardised outcome measure was noted as a significant barrier to implementing the MCN because it made it difficult to demonstrate the MCN’s worth to funders and prevented evaluation against agreed standards. It also presented challenges for the MCN to agreeing how success should be measured.

### People

Despite sharing the MCN’s vision for children’s palliative care, participants varied in their awareness of the MCN and its work. Several potential phase 2 participants excluded themselves from the study because they felt unable to say anything about the MCN, and many participants in both phases of the study were not clear about the specific purpose or role of the MCN.

The small number of specialist palliative care professionals and community nursing teams in the region were identified by numerous participants in phase 1 as a key barrier to the development of a 24/7 community-based service model, which was a core goal for the MCN.

*“There are areas of our geographical patch that don’t have good access to community nursing. It’s not about the skill of the people involved. It’s purely about their presence, their availability.” (Phase 2, participant #39)*

The shortage of specialist palliative care staff was recognised to be a national problem; however, there were more mixed views amongst participants from both phases about whether more specialist palliative care professionals were needed, or whether the solution was about working differently to ensure community nurses and others were able to access the specialist expertise available.

### Infrastructure

The limited and short-term nature of funding for the MCN was identified by many as a barrier to effective implementation, as was the fact that the MCN relied on the commitment of key individuals who were not MCN funded.

*“We’re continually having to look at getting that funding on an annual basis [and that process] takes away from the work that we’re doing.” (Phase 1, participant #10)*

Insufficient funding was a cross-cutting issue, impacting on the MCN’s ability to address barriers related to technology, training, service development and staffing. It also meant change was slow to be implemented, and individual and organisational engagement suffered because barriers were perceived by member organisations as being too challenging to overcome.

The size and geographical diversity of the region was another important barrier. Participants from organisations on the geographical periphery of the region were concerned that the MCN’s resources may not reach them, and also reported difficulties engaging with meetings and events that were held centrally. Some participants were concerned that if care was standardised across the region it wouldn’t be appropriate for some locations with local idiosyncrasies.

*“One model that fits all [would be] a real challenge because […] the service pattern and structure in each is place is different.” (Phase 1, participant #8)*

The appointment of the MCN’s full-time coordinator was named by nearly all participants as the most important enabler for formalising the MCN’s governance structures and driving forward the MCN’s goals. The role also provides the MCN with a single site of contact and liaison point to raise awareness and keep members updated.

### Technology

The MCN’s hubs and spokes use different patient record systems and there is no technological solution to share these data. This was identified as a cross-cutting barrier to implementing the MCN, impacting on its ability to improve access to specialist palliative care, increase co-ordination and collaboration between services, and an impediment to the MCN’s plans to create a specialist 24/7 service, which is reliant on health professionals providing telephone advice or care for children they do not know.

*“The classic is if the GP has started long acting morphine [and] I can’t find that [in their records] because [the GP uses a different system]. You’re reliant on people being able to grab you and phone you [so] it’s not a robust system.” (Phase 2, participant #46)*

Although data incompatibility is a known issue impeding joined-up care nationally, participants also stated it impeded the evaluation of the MCN due to the different information that is recorded by different organisations, the differences in how the same information is recorded, and the different functionalities for searching and extracting data. These issues were also highlighted by participants in relation to a pilot data collection process carried out by the MCN to understand hospice provision. Participant feedback suggests that some organisations will find it difficult to use a standardised data collection template and may worry that their provision is not being adequately captured. Participants also stated there is also a resource implication for organisations who have to manually search for information.

During this study, the MCN had secured funding for a virtual community of practice focused on improving the transition of children into adult services. Several phase 1 participants believed this could facilitate more accessible training, peer support and collaboration, and sharing of expertise and best practice. Although not yet implemented, this could be a potential enabler for the MCN in the future.

### Culture

Many participants in both phases talked about the impact of the diversity in member organisations: each has different priorities, funding structures, practices, and governance processes. The differences between the NHS and hospice organisations at the centre of the MCN – in terms of scale, models of care, specific populations served (e.g. different transition points into adult services, and security of funding) – were specifically highlighted as a key barrier to developing a co-ordinated MCN with shared governance, data, resources and care pathways. These differences also contributed to the uncertainty expressed by participants about the MCN.

*“I think there are issues around the definition […] you know, how the term palliative care is used in different places […] I just think that if we’re talking about specialist palliative care we need to make sure we’re talking about specialist palliative care consistently.” (Phase 1, participant #6)*

As highlighted previously, the limited amount of funding for the MCN was identified as a barrier to its development. For some participants, this reflected the fact that funding children’s palliative care is not a priority for healthcare commissioners.

*“Children’s and young people’s palliative care […] hasn’t been funded properly by the NHS for goodness knows how long” (Phase 2, participant #35)*

Several participants, principally those in phase 1, believed that the lack of sufficient funding for palliative care more generally placed constraints on what member organisations were able and willing to offer to the MCN, in terms of sharing and expanding resources and expertise.

*“One of the barriers is that there’s not enough funding to go around, and then that creates challenges and competition […] between different organisations.” (Phase 1, focus group 1 participant)*

### Processes and procedures

Participants in phase 1 generally felt that the MCN has a clear governing structure that includes all the hub and spoke organisations that make up the MCN and has a more formalised approach compared with the informal network that existed previously.

*“It has changed. It feels a bit more formalised now, a bit more structured; [there are] agendas and actual projects rather than just a forum that people went to.” (Phase 1, focus group 2 participant)*

This opinion was not unanimous, however: one participant in phase 1 referred to the MCN as a ‘talking shop’ and several other participants expressed concerns about the changeable and partial involvement of organisations in the MCN’s governance structures. Some participants in both phases also expressed uncertainty about whether the MCN itself had led to formal changes in the provision of palliative care, although their beliefs about why differed.

When asked what had been achieved, many participants in both phases talked about the MCN’s role in setting standards for palliative care in the region, including guideline development and endorsement. However, the varied knowledge and participation in the MCN across the region is likely to affect adoption of these, and as one participant noted, there is no legal requirement or agreed responsibility that members will use them.

Training provision by the MCN, which includes an annual conference and various study days, was positively evaluated by a number of participants who commented on the impact it has had on their practice, both from the training provided and the opportunity to learn from others working in children’s palliative care.

However, several participants, particularly those in phase 2, believed that the training offer was limited and needed to be expanded. There was evidence that staff in less clinical roles or those in services that provided universal services may not be benefiting, risking a widening of the skills gap between those already engaged in palliative care and the MCN, and others that are not.

*“I just felt it was all attended by people who were in that world. I didn’t really feel it was aimed at [non-clinical staff].” (Phase 2, participant #40)*

## Discussion

The study identified key features that aided development of an MCN for children’s palliative care, including having a funded MCN co-ordinator, a committed leadership team, a governance structure that fosters collaboration, and a shared vision for the future. Some of these factors have been identified as influencing MCN implementation in other contexts (see Table 1) [19]. However, the study found that these features alone, which have been identified in previous research as enablers for MCN success [19], were not sufficient to overcome the cross-cutting barriers that were impeding the MCN’s development. These included limited funding for the MCN and children’s palliative care generally, having no shared technology, the lack of standards and limited evidence base for children’s palliative care, and the shortage of community nursing and specialist palliative care staff. These barriers, some of which have also been identified as pertinent to adult palliative care networks [33], were found to impact on the MCN’s ability to agree and implement changes and evaluate success, and consequently on the extent of participation in the MCN amongst those who work in MCN member organisations.

Other implementation research exploring implementation of new innovations also highlights the significance of first-order problems [34], such as interoperability of information systems, and how they lead to second-order problems, such as developing workarounds to share patient data; and third-order problems, such as member disengagement. However, it is recognised that addressing first-order problems is not always possible when implementing new practices into existing infrastructures and organisations [34], and the cross-cutting barriers we identified are likely to hinder the development of other children’s palliative care networks, certainly within countries with established healthcare systems [19, 35, 36] and varying models of palliative care [33].

Understanding how these features of children’s palliative care influence MCN development can nevertheless inform planning of new networks and reduce the risk of associated problems that impact on member participation, which research consistently shows is not static and requires continuous work to sustain [25, 34, 37]. Local champions (individuals who work for MCN organisations and are involved in work to drive MCN implementation within their organisation) [38] could prove useful here although there is a lack of research about their role in children’s palliative care.

Agreeing the definition and model of children’s palliative care and ensuring there is clarity about this across organisations are also essential because of the different definitions and models of care that currently exist in the region [39, 40]. Although in this study there was a shared vision for equitable and around-the-clock palliative care across the region, there was also uncertainty about what this would look like, both in terms of service provision and who would be eligible. This was in part due to the holistic definition of paediatric palliative care adopted by the MCN, which whilst aligned with the World Health Organisation’s definition and the holistic offering of children’s hospices [41], generated uncertainty about what the MCN was trying to achieve. At the same time, the focus on developing specialist palliative care services, such as the development of palliative medicine teams in the local children’s hospitals, led to concerns about how other palliative care provision would be captured as part of the MCN, and the relevance of the MCN’s resources, e.g. training, for organisations providing universal services, such as social care and education.

In this study, training was generally well received by MCN members and offers networks an important mechanism through which to demonstrate the value of an MCN and keep member organisations engaged and connected with one another, as well as improve clinical practice. However, networks need to ensure its training meets the diverse needs of its members and is accessible across a network [42–44]. Virtual learning networks (e.g. Project ECHO) could play an important role, and evaluations of adult palliative care ECHO networks have reportedly increased staff knowledge and confidence, and improvements in clinical practice [45–47].

Robust evaluations of MCNs are particularly important because of the limited evidence in children’s palliative care. However, in this study the cross-cutting barriers to developing the MCN highlighted in this study have impeded plans for evaluation, and other regions are likely to experience similar challenges because of the lack of standards and care pathways, and the resulting lack of agreed outcomes and robust measurement tools [48]. There were, nonetheless, missed opportunities to demonstrate early successes, for example measuring uptake of newly endorsed guidelines, such as a new guideline on rapid transfers for end-of-life care. It is important that, in the absence of appropriate measurement tools, networks make use of process as well as outcome data to assess potential changes in the delivery of palliative care, and to understand issues surrounding implementation [49]. MCNs may also benefit from drawing on appropriate implementation frameworks to help plan and evaluate new services and practice changes [21, 23, 27, 50, 51]. For example, in this study the socio-technical systems framework (SST) facilitated an understanding of the cross-cutting nature of the identified key barriers.

### Study strengths and limitations

We used appropriate qualitative methods with a purposive sampling strategy to ensure a diverse sample. A clear and iterative analytical process was followed involving more than one researcher to ensure findings were credible and trustworthy.

This study focused on a single hub-and-spoke MCN for children’s palliative care and, therefore, the findings may not be transferable to other specialties or those using other models. There was also limited representation from organisations on the MCN’s periphery, although concerns relating to geography were raised by participants from umbrella organisations that covered those localities.

## Conclusions

Some of the key features of children’s palliative care can act as barriers to early development of an MCN and to implementing practice changes. Managing expectations and raising awareness, providing accessible and relevant training, and sharing early achievements can help to maintain member engagement, which is crucial to a network’s success and requires continuous attention. Robust evaluation of children’s palliative care networks is also essential and should make use of process as well as outcome data to better understand issues surrounding implementation, as well as potential changes in the delivery of palliative care.

## Supplementary Information

**Additional file 1.** Study Topic Guides.

## Data Availability

Because individuals involved in the study could be identified through them, the datasets generated and analysed for the current study are not publicly available, but are available from the corresponding author on reasonable request.

## Abbreviations

ECHO: Extension for Community Healthcare Outcomes
MCN: Managed Clinical Network
NPT: Normalization Process Theory
SST: Socio-technical Systems Theory
WHO: World Health Organization

## References

[1] WHO (2020). WHO Definition of Palliative Care.

[2] Hain R, Wallace A (2008). Progress in palliative care for children in the UK. Paediatr Child Health.

[3] Craft A, Killen S (2007). Palliative care services for children and young people in England. An Independent review for the Secretary of State.

[4] Fraser L, Gibson-Smith D, Jarvis S (2020). ‘Make every child count’: estimating current and future prevalence of children and young people with life-limiting conditions in the United Kingdom.

[5] Noyes J, Edwards RT, Hastings RP (2013). Evidence-based planning and costing palliative care services for children: novel multi-method epidemiological and economic exemplar. BMC Palliat Care.

[6] Hain R, Devins M, Hastings R (2013). Paediatric palliative care: development and pilot study of a ‘directory’ of life-limiting conditions. BMC Palliat Care.

[7] Joy I (2005). Valuing short lives: children with terminal conditions (a guide for donors and funders).

[8] Taylor J, Booth A, Beresford B, et al. Specialist paediatric palliative care for children and young people with cancer: A mixed methods systematic review. Palliat Med. 2020; In Press. 10.1177/0269216320908490 PMC7243084 32362212

[9] Hunt A, Coad J, West E (2013). The big study for life-limited children and their families: final research report.

[10] Constantinou G, Garcia R, Cook E (2019). Children's unmet palliative care needs: a scoping review of parents' perspectives. BMJ Support Palliat Care.

[11] Knapp C, Woodworth L, Wright M (2011). Pediatric palliative care provision around the world: a systematic review. Pediatr Blood Cancer.

[12] Connor S, Bermedo M, editors. Global atlas of palliative Care at the end of life. London: Worldwide Palliative Care Alliance; 2014.

[13] Chambers L, Goldman A, et al. Together for Short Lives. A Guide to Children’s Palliative Care. Bristol; 2018. https://www.togetherforshortlives.org.uk/wpcontent/uploads/2018/03/TfSL-A-Guide-to-Children%E2%80%99s-Palliative-Care-Fourth-Edition-5.pdf.

[14] Baker CD, Lorimer AR (2000). Cardiology: the development of a managed clinical network. BMJ.

[15] Department of Health (2016). Our Commitment to you for end of life care: The Government Response to the Review of Choice.

[16] National Institute for Health and Care Excellence (NICE) (2016). End of life care for infants, children and young people with life-limiting conditions: planning and management. NICE guideline [NG61].

[17] Together for Short Lives (2017). Setting up a Managed Clinical Network in Children’s Palliative Care.

[18] NHS Lothian (2020). Managed Clinical Networks.

[19] Brown BB, Patel C, McInnes E (2016). The effectiveness of clinical networks in improving quality of care and patient outcomes: a systematic review of quantitative and qualitative studies. BMC Health Serv Res.

[20] Addicott R, McGivern G, Ferlie E (2006). Networks, organizational learning and knowledge management: NHS Cancer networks. Public Money Manage.

[21] Leeds University Business School (2019). Socio-Technical Systems Theory.

[22] Clegg CW, Robinson MA, Davis MC (2017). Applying organizational psychology as a design science: A method for predicting malfunctions in socio-technical systems (PreMiSTS). Des Sci.

[23] Davis MC, Challenger R, Jayewardene DN (2014). Advancing socio-technical systems thinking: a call for bravery. Appl Ergon.

[24] May C, Finch T (2009). Implementing, embedding, and integrating practices: an outline of normalization process theory. Sociology.

[25] Murray E, Treweek S, Pope C (2010). Normalisation process theory: a framework for developing, evaluating and implementing complex interventions. BMC Med.

[26] May C, Rapley T, Mair FS (2015). Normalization Process Theory On-line Users’ Manual, Toolkit and NoMAD instrument.

[27] May CR, Cummings A, Girling M (2018). Using normalization process theory in feasibility studies and process evaluations of complex healthcare interventions: a systematic review. Implement Sci.

[28] Elrod JK, Fortenberry JL (2017). The hub-and-spoke organization design: an avenue for serving patients well. BMC Health Serv Res.

[29] Krueger R, Casey M (2009). Focus groups: a practical guide for applied research.

[30] Atkins L, Francis J, Islam R, et al. A guide to using the Theoretical Domains Framework of behaviour change to investigate implementation problems. Implement Sci. 2017;12(1):77.https://www.ncbi.nlm.nih.gov/pmc/articles/PMC5480145/. 10.1186/s13012-017-0605-9 PMC5480145 28637486

[31] Braun V, Clarke V (2006). Using thematic analysis in psychology. Qual Res Psychol.

[32] NVivo qualitative data analysis software [program]. 12 version, 2018.

[33] McKinlay E, Esplin J, Howard-Brown C, et al. Implementing a managed clinical network in a small country: a New Zealand case study. Int J Healthc Manage. 2020:1–8. 10.1080/20479700.2020.1713536.

[34] Greenhalgh T, Wherton J, Papoutsi C (2017). Beyond adoption: a new framework for theorizing and evaluating nonadoption, abandonment, and challenges to the scale-up, spread, and sustainability of health and care technologies. J Med Internet Res.

[35] McInnes E, Haines M, Dominello A (2015). What are the reasons for clinical network success? A qualitative study. BMC Health Serv Res.

[36] Manns BJ, Wasylak T (2019). Clinical networks: enablers of health system change. CMAJ.

[37] Taylor J, Coates E, Brewster L (2015). Examining the use of telehealth in community nursing: identifying the factors affecting frontline staff acceptance and telehealth adoption. J Adv Nurs.

[38] Miech E, Rattray N, Flanagan M (2018). Inside help: an integrative review of champions in healthcare-related implementation. SAGE Open Med.

[39] Fraser LK, Jarvis S, Moran N (2015). Children in Scotland requiring palliative care: identifying numbers and needs (the ChiSP study).

[40] Mitchell S, Morris A, Bennett K (2017). Specialist paediatric palliative care services: what are the benefits?. Arch Dis Child.

[41] World Health Organization (2017). WHO Definition of Palliative Care for Children.

[42] Atout M. Experience of nurses who work with children with palliative care needs: A mixed-method systematic review. Palliat Support Care. 2019:1–13. 10.1017/S1478951519000956 [published Online First: 2019/11/28]. 10.1017/S1478951519000956 31774390

[43] Taylor J, Aldridge J (2017). Exploring the rewards and challenges of paediatric palliative care work - a qualitative study of a multi-disciplinary children's hospice care team. BMC Palliat Care.

[44] Brock KE, Cohen HJ, Sourkes BM (2017). Training Pediatric Fellows in Palliative Care: A Pilot Comparison of Simulation Training and Didactic Education. J Palliat Med.

[45] White C, McIlfatrick S, Dunwoody L (2019). Supporting and improving community health services-a prospective evaluation of ECHO technology in community palliative care nursing teams. BMJ Support Palliat Care.

[46] Píriz AG (2018). Technology for improving accessibility of end-of-life care: Extension for Community Healthcare Outcomes Project. Curr Opin Support Palliat Care.

[47] McBain RK, Sousa JL, Rose AJ (2019). Impact of Project ECHO Models of Medical Tele-Education: a Systematic Review. J Gen Intern Med.

[48] Widger K, Medeiros C, Trenholm M (2018). Indicators used to assess the impact of specialized pediatric palliative care: a scoping review. J Palliat Med.

[49] Moore GF, Audrey S, Barker M (2015). Process evaluation of complex interventions: Medical Research Council guidance. BMJ.

[50] Serhal E, Arena A, Sockalingam S (2018). Adapting the consolidated framework for implementation research to create organizational readiness and implementation tools for project ECHO. J Contin Educ Heal Prof.

[51] Harvey G, Kitson A (2016). PARIHS revisited: from heuristic to integrated framework for the successful implementation of knowledge into practice. Implement Sci.

