# Supplementary material for: Development of a managed clinical network for children’s palliative care – a qualitative evaluation
Source: BMC Palliat Care. 2021 Jan 22;20:20. doi: 10.1186/s12904-021-00712-7 (PMC7824916; doi:10.1186/s12904-021-00712-7)
Supplement: Supplementary file 1 — Additional file 1. Study Topic Guides. [file 12904_2021_712_MOESM1_ESM.zip › MCN Eval P1 Is Topic GuideR3.pdf]

# Implementation of a Managed Clinical Network for Children's Palliative Care – A Qualitative Evaluation

## <u>Phase One Topic Guide – INTERVIEW</u>

## 1. Background information

Job role: role and profession, organisation, duration in post and profession

**YHCPCN / MCN role: specific role / responsibilities re: network, duration of involvement**

## 2. Defining and describing the network

- **What is the Managed Clinical Network?**
- What are the aims / goals for the Managed Clinical Network?
- How does the MCN differ to the YHCPCN?
- Prompts: Which organisations are part of the Managed Clinical Network? Who is responsible for implementation?

## 3. Perceived impacts

- What does the Network mean for the funding / co-ordination / delivery of palliative care in the region (e.g. changes, processes, impacts)?
- **How will the Network impact on member organisations and services / clinicians / children and families?**
- Prompts: 24/7 care? Access / equity of care? Positive impacts or potential positives?

## 4. Implementation

- What's been done so far in terms of implementing the Network?
- What's still to do?
- **In terms of implementing the Network as planned, what barriers have you encountered / do you think you will encounter?**
- **What has helped or may help to implement the Network as planned?**
- How have member organisations and their staff responded so far?
- Prompts: What is the role and barriers of information systems?
