# Supplementary material for: Development of a managed clinical network for children’s palliative care – a qualitative evaluation
Source: BMC Palliat Care. 2021 Jan 22;20:20. doi: 10.1186/s12904-021-00712-7 (PMC7824916; doi:10.1186/s12904-021-00712-7)
Supplement: Supplementary file 1 — Additional file 1. Study Topic Guides. [file 12904_2021_712_MOESM1_ESM.zip › MCN Eval P2 Topic GuideR3.pdf]

Implementation of a Managed Clinical Network for Children's Palliative Care – A Qualitative Evaluation

Phase Two Topic Guide

1. Background information

Job role: role and profession, organisation, duration in post and profession, % of role providing palliative care and / or providing care for children and young people with LLCs

2. Knowledge of the Network

- **What do you know about the Managed Clinical Network?**
  - What are the aims / goals?
  - What does the Network do (e.g. host events and training, provide care etc.)?
- **What has been done to achieve these aims?**
- **What communications have you received?**

- Other information known about the network
  - Who is involved in the Network?
  - Who is the lead for your organisation?
  - How and when did you find out about the Network?

(If they have no knowledge of the Network ask about their knowledge of how palliative care for children is co-ordinated / provided in the region and who does this.)

3. Involvement in the Network

- How have you / your organisation been involved with the Network?
  - Prompts: attended events e.g. conference, received training e.g. module for CPC, sought input / advice, spoken to individual members of the Network, worked with new people
- How has your / your organisation's involvement impacted on you and your work (i.e. what are the benefits and drawbacks) or changed the way you work?
  - Prompts: increased knowledge, confidence, more collaboration, better care for children, more work, working with children not known to them
- Tell us about how your colleagues and the organisation is involved in the Network?
- What are the changes (benefits and drawbacks) for your organisation?
- How important is the Network to you and to your organisation?

(Again if they have no knowledge about the Network ask about how they access events, training, palliative care expertise in their role and from whom.)

4. Implementation of the Network

- **What are the barriers to achieving the Network's aims (if given)?**
  - Prompts: Funding, Governance, Geography, Organisational factors, Data
- Have there been particular factors preventing you and your organisation from being more involved in the Network?
  - Prompts: Funding, Governance, Geography, Organisational factors, Data
- What solutions are there to these barriers/factors?
  - Prompts: Improved data management and sharing, Improved training, Improved leadership/governance, More involvement from senior leadership
- Can information systems help to facilitate the transition?
- If the barriers are addressed, how will the Network impact on you, your organisation and the children and families you support in the future?
